# Can the body composition of crossbred dairy cattle be predicted by equations for beef cattle?

**DOI:** 10.5713/ajas.17.0876

**Published:** 2018-04-12

**Authors:** Maria Luciana Menezes Wanderley Neves, Evaristo Jorge Oliveira de Souza, Robson Magno Liberal Véras, Sebastião de Campos Valadares Filho, Marcos Inácio Marcondes, Gabriel Santana da Silva, Lígia Maria Gomes Barreto, Marcelo de Andrade Ferreira, Antonia Sherlânea Chaves Véras

**Affiliations:** 1 Department of Animal Science, Federal Rural University of Pernambuco, UFRPE, Recife, Pernambuco 52171900, Brazil; 2 Serra Talhada Academic Unit, UAST/UFRPE, Serra Talhada, Pernambuco, Caixa Postal 063, 56903970, Brazil; 3 Garanhuns Academic Unit, UAG/UFRPE, Garanhuns, Pernambuco 55292270, Brazil; 4 Department of Animal Science, Federal University of Viçosa, Viçosa, Minas Gerais 36570900, Brazil; 5 Federal University of Sergipe, Nossa Senhora da Glória, Sergipe 49680000, Brazil

**Keywords:** Chemical Composition, Physical Composition, Rib Section Cut

## Abstract

**Objective:**

The aim of the study was to evaluate the efficiency of the Hankins and Howe (HH46), Valadares Filho (V06), and Marcondes (M12) equations for predicting the physical and chemical composition of dairy crossbred bulls carcasses, as well as the chemical composition of their empty bodies.

**Methods:**

This study was conducted using 30 dairy crossbred bulls. One group of five animals was slaughtered at the beginning of the experiment, and the remaining were slaughtered 112 days later. Animals were distributed in a completely randomized design into treatments consisting different levels of concentrate (0%, 17%, 34%, 51%, and 68%). The physical and chemical compositions of the cattle were obtained from the right half of the carcass and using samples taken between the 9th and 11th ribs of the left half of the carcass. The estimated and experimentally determined values were compared using the correlation and concordance coefficient, as well as the mean square error of prediction (MSEP) and its components.

**Results:**

The HH46 equations were better at estimating the amount of muscle plus fat in the carcass. The amount of bone in the carcasses could not be well estimated by the HH46 and M12 models. The M12, HH46, and V06 equations were worst at estimating the amounts of protein, ether extract, and water in the carcass, respectively. In the empty body, the amounts of protein and water were well estimated by the HH46 equations. Protein, ether extract, and water were accurately estimated by the V06 equations, and ether extract by the M12 equations.

**Conclusion:**

The physical and chemical composition of dairy crossbred bull carcasses, as well as the chemical composition of their empty bodies, can be predicted using the equations tested here. The amount of bone in these carcasses could not be accurately predicted.

## INTRODUCTION

To reduce the time and cost spent on the complete dissection of cattle carcasses, Hankins and Howe [1] proposed carcass sampling, in which a section between the 9th and 11th ribs is used to estimate the physical and chemical compositions of bovine carcasses. However, these equations were developed using data from *Bos taurus taurus*, which has a different body composition than zebu or crossbred [2], and the direct methods have been progressively abandoned for indirect methods [3].

Work by Marcondes et al [4], Paulino et al [5], and Silva et al [6] has shown that the equations of Hankins and Howe [1] were not fully applicable for zebu, and suggested new equations and approaches [7,8]. These new equations were developed using data from zebu and crossbred cattle and validated for Nellore cattle [9], which differ from Holstein×Gir crossbred cattle.

The use of crossbred Holstein×Gir for milk production is common in tropical regions. This cross produces animals that are more tolerant to tropical conditions while maintaining good productivity. Thus, male calves from Holstein×Gir crosses are an important alternative meat source in Brazil, as well as in other parts of the world. Despite the importance of this genetic group, there is little available data on Holstein×Gir crossbred cattle [10–14]. Here, we aim to test the applicability of available equations for estimating the physical and chemical composition of crossbred dairy cattle.

## MATERIALS AND METHODS

### Ethical considerations

This work was conducted with the approval of the Animal Use Ethics Committee of Federal Rural University of Pernambuco (UFRPE), Recife, Pernambuco, Brazil (Permit Number: 23082.015634/2012–41).

### Animal, diets and experimental treatments

Thirty crossbred dairy bulls, with an average initial body weight (BW) of 339.1 (standard deviation = 35.4 kg) were used. First, animals were weighed, identified, treated against internal and external parasites, and supplemented with injectable vitamin A, D, and E.

After a 30 day adaptation period, in which animals were fed the same diet (80:20, forage:concentrate), five animals were randomly chosen as the reference group. The remaining animals were randomly distributed in a completely randomized design consisting five treatments (either 0%, 17%, 34%, 51%, or 68% of concentrate [dry matter basis]), confined for 112 days, and slaughtered.

Diets were formulated according to National Research Council [15] for a weight gain of 1 kg/d (Table 1). Feeding was carried out twice per day as a total mixed ration and adjusted to allow leftovers of between 5% and 10% of the quantity supplied, except for animals of the maintenance group (0% concentrate, dry matter intake restricted for 1.5% BW). Water was permanently available.

### Slaughter and samplings

At the end of the experiment, the animals were slaughtered after 16 h of fasting. Procedures followed Instruction No. 3 (17 Jan 2000) of the Ministry of Agriculture, Livestock and Food Supply, which regulates humanitarian slaughter [16].

To determine the empty body weight (EBW), the gastrointestinal tract was emptied, washed, and its weight added to the remaining parts of the empty body (organs, viscera, internal fat, carcass, head, leather, tail, feet, and blood). To obtain the cold carcass weight, the carcass of each animal was divided into two half carcasses, which were weighed and then cooled in a cold chamber at 4°C for 24 h. From the left half of the carcass, the section between the 9th and 11th ribs (rib_9–11_), according to the methodology described by Hankins and Howe [1], was collected, weighed, dissected, and the proportions of muscle, adipose, and bone tissue contained therein were determined. Bone tissue was also separated from the other tissues of the right half of the carcass, which were weighed to further evaluate the physical composition of the carcass.

Blood was collected separately during bleeding, and blood samples were pre-dried at 55°C to 60°C for 72 h. Organs, viscera, and internal fat were ground in a milling cutter in order to obtain a composed sample, as well as muscle plus fat from the right half carcass and the rib_9–11_. Leather samples were chopped, and tail, head, feet, and bones samples were sawed.

### Laboratory analysis

To evaluate the fat dry matter (FDM), and water content, organs and viscera, muscle and fat, leather, bone, tail, head, and limbs were dried in stove at 105°C (method 24.002 [17]).

Later, a pre-degreasing was performed on the samples through successive washings with petroleum ether, to obtain the pre-defatted dry matter (PDDM). Then, samples were ground in a ball mill for further analysis and for quantification of the ether extract (EE, method 24.005 [17]) and nitrogen (N, method 24.038 [17]) contents. The protein content (P) was calculated by multiplying the nitrogen content by 6.25 [17]. Fat removed in the pre-degreasing was calculated as the difference between FDM and PDDM and added to the results obtained for the residual EE in PDDM for determination of the total fat content.

### Evaluation of equations

The equations proposed by Hankins and Howe [1] (HH46), Valadares Filho et al [8] (V06), and Marcondes et al [7] (M12) were evaluated. The chemical composition of the rib_9–11_, as well as other parameters, were used to design these equations (Table 2).

### Data used in the experiment

The data used to test the equations are shown in Table 3 and were evaluated in their absolute weight (kg), according to the recommendations of Costa e Silva et al [9].

### Statistical analysis

The body composition estimated by the equations of HH46, V06, and M12 were compared with the values observed using the regression model:

$$\text{Y}=\beta_{0}+\beta_{1}\times \text{X}$$

Where X = predicted value; Y = observed value; β_0_ and β_1_ = intercept and inclination coefficient of the linear equation, under the following statistical hypothesis:

$${\text{H}}_{0}:\beta_{0}=0\;\text{and }{\text{H}}_{0}:\beta_{1}=1\;\text{and }{\text{H}}_{\text{a}}:\text{not }{\text{H}}_{0}$$

If the null hypotheses were not rejected, the prediction model accurately estimated the evaluated body composition.

The efficiency of the prediction models was also evaluated using the mean square error of the prediction (MSEP) and its components [18]:

$$\text{MSEP}=\text{SB}+\text{MaF}+\text{MoF}=1/\text{n }\Sigma_{\text{i}=1}{\left({\text{x}}_{\text{i}}-{\text{y}}_{\text{i}}\right)}^{2}$$

SB = (x–y)^2^, MaF = (s_x_–s_y_)^2^, MoF = 2s_x_s_y_(1–r), where x are the predicted values; y are the observed values; MSEP is the mean square error of the prediction; SB is the squared bias; MaF is the magnitude of random fluctuation component; MoF is the model of random fluctuation component; s_x_ and s_y_ are the standard deviations of the predicted and observed values, respectively; and r is the Pearson linear correlation coefficient between the predicted and observed values. Accuracy and precision of the model were evaluated by estimating the correlation and concordance coefficient (CCC) or the reproducibility index, described by Tedeschi [19]. Models were statistically evaluated using the Model Evaluation System (3.1.16) software (http://nutritionmodels.com/mes.html).

## RESULTS

### Carcass physical composition

The equation proposed by HH46 was best at estimating the composition of the muscle tissue plus adipose tissue in the carcass when compared with M12 when the CCC was observed (Table 4). The intercept and inclination of the M12 equation presented problems (p<0.05), whereas the parameters of the HH46 equation were accurately estimated (p>0.05). The amount of muscle plus fat was underestimated (Table 4) by the M12 equation.

The HH46 and M12 equations returned low CCC values when predicting the amount of bone tissue in the carcass (Table 4), and were unable to adequately estimate the β_0_ and β_1_ parameters (p<0.05). Both equations overestimated the amount of bone tissue in the carcass for the majority of the evaluated samples (Table 4).

### Carcass chemical composition

When estimating the chemical composition of the carcass, the V06 equations returned the best CCC values for all chemical components analyzed (Table 5).

All of the tested models performed well when estimating the amount of P in the carcass (all with high CCC and low MSEP values). However, the M12 model have problems with intercept and slope was unable to adequately estimate the equation parameters (p<0.05).

When estimating the EE, the M12 and V06 returned about equal CCC and MSEP values. Using the HH46 equation returned high CCC and MSEP values, and the coefficient of inclination was not properly estimated (p<0.05). Most of the EE concentration was overestimated by the HH46 equation (Table 5).

When estimating the amount of water in the carcass, the V06 equation returned a higher CCC value and lower MSEP value than the other equations. However, the intercept and the slope were not well estimated using V06 (p<0.05). On the other hand, this problem was not observed for the HH46 and M12 equations, which both returned high CCC values (Table 5). The M12 equation returned a lower MSEP value than the HH46 equation. However, 55% of the MSEP of the HH46 equation’s were found in the MoF, which means that most of the deviation was not due to intrinsic errors in the β_0_ and β_1_ parameters of the linear equation.

### Empty body chemical composition

When estimating the chemical components in the empty body, all of the tested equations returned high CCC values (Table 6).

The MSEP was similar between M12 and V06 equations for EE and P, with most of the MSEP relative to MoF, indicating that most of this error was unrelated to the equation.

When estimating water content, the MSEP of the V06 equation were smaller than the M12 equation. The highest proportion MSEP of the M12 model was associated with the deviation in the β_0_ of the equation, this was supported by the SB value for this equation. Thus, V06 equation were better, because most of the MSEP was not relative to the model (MoF = 39.39).

## DISCUSSION

### Carcass physical composition

The performance of the HH46 equation when estimating the amount of muscle plus fat in the carcass might be connected to the antagonism of these two components, leading to a positive result when these two variables are added together (Table 4). HH46 developed equations for calves, heifers, and composed equations. Here, we used the equations developed for calves, whose fat deposition occurs later in heifers and in bulls. Because for this difference in the deposition of tissues between the genders, some studies have reported that these equations overestimate the amount of fat [4,9] and undervalue the amount of muscle [4].

The introduction of internal fat in the M12 equation may have interfered in its efficiency at estimating muscle plus fat in crossbred dairy bulls, since this animal category tends to store dietary energy excess in the form of internal fat [20].

The low efficiency of the HH46 and M12 equations at estimating the amount of bone tissue may be related to the genetic group used in the study. Costa e Silva et al [9] found that the HH46 and M12 equations adequately estimated the amount of bone tissue in Nellore cattle. However, Goulart et al [21] observed differences in the proportion of bone in animals from distinct genetic groups.

### Carcass chemical composition

None of the evaluated models could adequately estimate all of the chemical components in the carcass (Table 5). However, we propose that either the HH46 or M12 equations can be used for predicting the water content of crossbred dairy cattle; the HH46 or V06 equations for estimating protein content; and the V06 or M12 equations for EE. Prados [22] has proposed that the V06 and M12 equations are suitable for estimating P, EE, and water in Holstein×Gir cattle carcass and that the HH46 equations were better predictors of P content.

### Empty body chemical composition

We found that the V06 equations are suitable for estimating EE and water in the empty bodies of crossbred dairy cattle (Table 6), which is in line with the findings of Prados [22]. In this study, the V06 equation was best at estimating the amount of protein in the empty body. However, Prados [22] and Costa e Silva et al [9] found that the M12 model performed best with crossbred dairy and Nellore cattle respectively. Thus, further studies should aim to resolve the best approach for estimating the chemical composition in the empty bodies of crossbred dairy cattle.

## CONCLUSION

In crossbred dairy bulls, the amount of fat plus muscle in the carcass can be estimated using the HH46 equations. However, the amount of bone tissue in the carcass cannot be estimated from the HH46 and M12 models. The M12, HH46, and V06 models were worst at estimating the amounts of protein, EE, and water in the carcass, respectively. In the empty body, the amount water are best estimated using the equation proposed by V06; protein, and EE, are best estimated by the V06 and M12 equations. Overall, we propose that for dairy crossbred bulls, the carcass physical and chemical composition (except bone content) and empty body chemical composition can be predicted using the equations tested in this work.

## Figures and Tables

**Table 1:** Chemical composition of experimental diets

Nutritional components	Concentrate levels (% DM)				

	0	17	34	51	68
Dry matter (g/kg of natural matter)	890.40	891.34	892.28	893.22	894.16
Organic matter (g/kg DM)	932.17	928.91	925.66	922.40	919.15
Crude protein (g/kg DM)	92.60	109.64	126.68	143.72	160.76
Neutral detergent fibre (g/kg DM)	708.05	616.10	524.15	432.20	340.25
Acid detergent fibre (g/kg DM)	284.80	245.89	206.97	168.06	129.15
Total carbohydrates (g/kg DM)	823.58	808.11	792.63	777.15	761.68
Non-fibre carbohydrates (g/kg DM)	115.53	192.00	268.48	344.96	421.43
Ether extract (g/kg DM)	23.42	25.17	26.92	28.67	30.42
Total digestible nutrients (g/kg DM)1)	563.46	574.41	635.24	678.69	732.37
Metabolizable energy (kcal/d)2)	203.71	207.67	229.66	245.37	264.78

DM, dry matter; TDN, total digestible nutrients.

1) Estimated by Silva et al [23].

2) ME = TDN×4.409×0.82.

**Table 2:** Equations used to estimate the body composition in bulls

Items	Equations		

	Hankins and Howe [1] (HH46)	Valadares Filho et al [2] (V06)	Marcondes et al [3] (M12)
Carcass physical composition			
% Muscle	%M_C_ = 16.08+0.8×%M_rib9–11_	-	%M_C_ = 57.33+0.2×%M_rib9–11_–1.39×VF
% Fat	%F_C_ = 3.54+0.8×%F_rib9–11_	-	%F_C_ = 0.689+0.3×%F_rib9–11_+1.177×%VF
% Bone	%B_C_ = 5.52+0.57×%B_rib9–11_	-	%B_C_ = 29.26+0.3×%B_rib9–11_–0.21×%CD–0.95×%VF
Carcass chemical composition			
% Protein	%P_C_ = 6.19+0.65×%P_rib9–11_	% P_C_ = 4.05+0.78×%P_rib9–11_	% P_C_ = 17.92+0.6×%P_rib9–11_–0.17×%CD
% Ether extract	%EE_C_ = 3.49+0.74×%EE_rib9–11_	%EE_C_ = 4.96+0.54×%EE_rib9–11_	%EE_C_ = 4.31+0.31×%EE_rib9–11_+1.37×%VF
% Water	%W_C_ = 16.83+0.75×%W_rib9–11_	%W_C_ = 34.97+0.45×%W_rib9–11_	%W_C_ = 48.74+0.28×%W_rib9–11_–0.017×EBW
Empty body chemical composition			
% Protein	-	% P_EBW_ = 4.96+0.76×%P_rib9–11_	% P_EBW_ = 10.78+0.47×P_rib9–11_–0.21×%VF
% Ether extract	-	%EE_EBW_ = 4.56+0.6×%EE_rib9–11_	%EE_EBW_ = 2.75+0.33×EE_rib9–11_+1.8×%VF
% Water	-	%W_EBW_ = 31.42+0.51×%W_rib9–11_	%W_EBW_ = 38.31+0.33×W_rib9–11_–1.09×%VF+0.5×OV

M_C_, muscle in the carcass; F_C_, fat in the carcass; B_C_, bone in the carcass; M_rib9–11_, muscle in the section between the 9th and 11th ribs; F_rib9–11_, fat in the section between the 9th and 11th ribs; B_rib9–11_, bone in the section between the 9th and 11th ribs; CD, carcass dressing in the empty body; P_C_, protein in the carcass; EE_C_, ether extract in the carcass; W_C_, water in the carcass; P_rib9–11_, P in the section between the 9th and 11th ribs; EE_rib9–11_, EE in the section between the 9th and 11th ribs; W_rib9–11_, water in the section between the 9th and 11th ribs; VF, visceral fat; EBW, empty body weight; OV, organs and viscera.

**Table 3:** Variables used to estimate the physical and chemical composition of the carcasses, as well as the chemical composition of the empty bodies

Items	Mean	SD	Maximum	Minimum
Empty body weight (kg)	361.78	83.98	528.81	224.44
Cold carcass weight (kg)	228.54	58.4	342.8	135.6
Organs and viscera (% EBW)	12.99	0.97	15.6	11.51
Visceral fat (% EBW)	4.17	1.28	7.51	1.95
Cold carcass yield (% EBW)	62.82	2.39	66.76	56.61
Muscle and fat in the carcass (%)	84.03	4.04	89.5	73.09
Bone in the carcass (%)	15.97	4.04	26.91	10.50
Crude protein in the carcass (%)	18.54	0.87	20.31	16.85
Ether extract in the carcass (%)	15.96	3.53	24.51	7.87
Water in the carcass (%)	60.23	2.79	66.85	54.63
Crude protein in the EBW (%)	19.26	0.96	21.2	16.66
Ether extract in the EBW (%)	16.17	3.34	24.47	10.49
Water in the EBW (%)	60.15	2.59	64.83	54.74
Muscle in the rib_9–11_ (%)	58.96	4.21	66.29	49.19
Fat in the rib_9–11_ (%)	19.46	7.4	32.63	6.96
Bone in the rib_9–11_ (%)	21.58	5.62	35.1	13.73
Crude protein in the rib_9–11_ (%)	18.88	1.6	22.43	16.33
Ether extract in the rib_9–11_ (%)	18.97	6.21	31.62	6.53
Water in the rib_9–11_ (%)	54.84	4.11	61.94	45

SD, standard deviation; EBW, empty body weight; rib_9–11_, section between the 9th and 11th ribs.

**Table 4:** Descriptive statistic of the relationships between the observed and predicted carcass physical composition values

Items	Muscle+fat			Bone		
	
	Obs	HH46	M	Obs	HH46	M
Mean (kg)	193.65	189.78	171.47	34.99	39.61	41.27
SD	55.63	54.48	46.04	6.76	8.46	7.93
Maximum	302.61	280.06	257.67	54.40	62.70	63.35
Minimum	104.93	110.46	100.26	24.95	25.03	27.82
r	-	0.98	0.99	-	0.38	0.45
CCC	-	0.98	0.89	-	0.31	0.33
Regression						
Intercept	-	2.90	−12.22	-	22.98	18.97
SE	-	6.68	4.53	-	5.66	6.03
p-value1)	-	0.668	0.012	-	<0.001	0.005
Slope	-	1.01	1.20	-	0.30	0.39
SE	-	0.03	0.03	-	0.14	0.14
p-value2)	-	0.882	<0.001	-	<0.001	<0.001
MSEP	-	107.31	611.64	-	93.92	98.61
SB	-	14.92	491.69	-	22.24	40.62
MaF	-	0.07	82.44	-	33.82	22.91
MoF	-	92.31	37.50	-	37.86	35.08

Obs, observed values; HH46, predicted values by the equations Hankins and Howe [1]; M, predicted values by the equations Marcondes et al [3]; SD, standard deviation; r, correlation coefficient; CCC, correlation and concordance coefficient; SE, standard error; MSEP, mean square error of prediction; SB, square bias; MaF, magnitude of random fluctuation; MoF, model of random fluctuation.

1) H_0_: b_0_ = 0.

2) H_0_: b_1_ = 1.

**Table 5:** Descriptive statistics of the relationships between the observed and predicted carcass chemical composition values

Items	Crude protein				Ether extract				Water			
		
	Obs	HH46	V06	M12	Obs	HH46	V06	M12	Obs	HH46	V06	M12
Mean (kg)	42.20	41.79	42.42	41.90	37.91	42.00	36.17	37.74	136.51	131.35	135.65	131.21
SD	10.20	9.37	9.30	8.96	16.56	18.80	15.04	15.41	30.89	30.26	32.55	29.09
Maximum	61.27	61.01	61.76	60.28	84.03	75.39	63.28	60.56	198.28	197.62	202.92	187.64
Minimum	25.11	26.77	27.55	27.34	12.25	12.56	12.81	15.21	87.46	85.82	85.22	84.44
r	-	0.99	0.98	0.98	-	0.94	0.94	0.93	-	0.98	0.99	0.99
CCC	-	0.98	0.98	0.97	-	0.91	0.93	0.93	-	0.97	0.99	0.97
Regression												
Intercept	-	−2.62	−3.49	−4.60	-	3.14	0.30	0.00	-	4.83	9.14	−1.62
SE	-	1.52	1.68	1.80	-	2.62	2.68	2.95	-	4.89	3.66	3.52
p-value1)	-	0.095	0.050	0.016	-	0.240	0.911	0.999	-	0.332	0.019	0.648
Slope	-	1.07	1.08	1.12	-	0.83	1.04	1.00	-	1.00	0.94	1.05
SE	-	0.04	0.04	0.04	-	0.06	0.07	0.07	-	0.04	0.03	0.03
p-value2)	-	0.050	0.057	0.009	-	0.005	0.566	0.953	-	0.945	0.028	0.054
MSEP	-	3.60	4.08	4.98	-	58.13	32.21	33.90	-	59.37	24.34	46.13
SB	-	0.17	0.05	0.09	-	16.78	3.03	0.03	-	26.63	0.74	28.09
MaF	-	0.45	0.50	1.06	-	10.15	0.35	0.00	-	0.01	3.82	2.28
MoF	-	2.98	3.53	3.82	-	31.20	28.83	33.87	-	32.73	19.79	15.76

Obs, observed values; HH46, predicted values by the equations Hankins and Howe [1]; V06, predicted values by the equations Valadares Filho et al [2]; M12, predicted values by the equations Marcondes et al [3]; SD, standard deviation; r, correlation coefficient; CCC, correlation and concordance coefficient; SE, standard error; MSEP, mean square error of prediction; SB, square bias; MaF, magnitude of random fluctuation; MoF, model of random fluctuation.

1) H_0_: b_0_ = 0.

2) H_0_: b_1_ = 1.

**Table 6:** Descriptive statistics of the relationships between the observed and predicted empty body composition values

Items	Crude protein			Ether extract			Water		
		
	Obs	V06	M12	Obs	V06	M12	Obs	V06	M12
Mean (kg)	69.30	69.19	67.40	60.43	59.88	62.05	216.20	213.81	209.77
SD	14.77	13.75	13.73	23.81	24.27	25.45	45.08	46.00	43.76
Maximum	97.36	97.99	96.48	112.98	103.44	99.21	311.61	311.35	302.25
Minimum	43.78	48.70	44.77	26.20	22.61	25.26	143.71	141.42	139.50
r	-	0.98	0.99	-	0.96	0.96	-	0.99	0.99
CCC	-	0.98	0.97	-	0.96	0.96	-	0.99	0.98
Regression									
Intercept	-	−3.50	−2.20	-	4.03	4.63	-	8.79	1.50
SE	-	2.90	2.31	-	3.34	3.26	-	5.73	4.70
p-value1)	-	0.236	0.350	-	0.238	0.167	-	0.136	0.752
Slope	-	1.05	1.06	-	0.94	0.90	-	0.97	1.02
SE	-	0.04	0.03	-	0.05	0.05	-	0.03	0.02
p-value2)	-	0.215	0.082	-	0.272	0.050	-	0.263	0.293
MSEP	-	9.14	10.07	-	45.12	50.69	-	46.93	67.31
SB	-	0.01	3.61	-	0.31	2.62	-	5.71	41.34
MaF	-	0.50	0.67	-	1.92	6.34	-	1.83	1.02
MoF	-	8.63	5.79	-	42.89	41.73	-	39.39	24.94

Obs, observed values; V06, predicted values by the equations Valadares Filho et al [2]; M12, predicted values by the equations Marcondes et al [3]; SD, standard deviation; r, correlation coefficient; CCC, correlation and concordance coefficient; SE, standard error; MSEP, mean square error of prediction; SB, square bias; MaF, magnitude of random fluctuation; MoF, model of random fluctuation.

1) H_0_: b_0_ = 0.

2) H_0_: b_1_ = 1.

## References

[1] Hankins OG, Howe PE (1946). Estimation of the composition of beef carcasses and cuts.

[2] Costa e Silva LF, Valadares Filho SC, Rotta PP, Valadares Filho SC, Costa e Silva LF, Gionbelli MP (2016). Prediction of body and carcass composition of beef cattle. Nutrient requirements of zebu and crossbred cattle.

[3] Al-Jammas M, Agabriel J, Vernet J, Ortigues-Marty I (2016). The chemical composition of carcasses can be predicted from proxy traits in finishing male beef cattle: a meta-analysis. Meat Sci.

[4] Marcondes MI, Valadares Filho SC, Paulino PVR (2009). Predicting body and carcass composition using the section between 9th and 11th ribs in Nellore cattle. R Bras Zootec.

[5] Paulino PVR, Costa MIL, Valadares Filho SC (2005). Validation of the equations proposed by Hankins and Howe for estimating the carcass composition of zebu cattle and development of equations to predict the body composition. R Bras Zootec.

[6] Silva FF, Valadares Filho SC, Ítavo LCV (2002). Intake, performance, carcass characteristics and biometric of gastrointestinal tract and internal organs of Nellore bulls receiving diets with different concentrate and protein levels. R Bras Zootec.

[7] Marcondes MI, Tedeschi LO, Valadares Filho SC, Chizzotti ML (2012). Prediction of physical and chemical body compositions of purebred and crossbred Nellore cattle using the composition of a rib section. J Anim Sci.

[8] Valadares Filho SC, Paulino PVR, Magalhães KA (2006). Nutritional requirement of zebu cattle and feed composition tables BR - CORTE.

[9] Costa e Silva LF, Valadares Filho SC, Detmann E (2013). Evaluation of equations to predict body composition in Nelore bulls. Livest Sci.

[10] Andrade DKB, Véras ASC, Ferreira MA (2008). Body composition and net requirements of macrominerals for gain of 5/8 crossbreed bulls under grazing in coastal area of Pernambuco state. R Bras Zootec.

[11] Andrade DKB, Véras ASC, Ferreira MA (2009). Body composition and net protein and energy requirements for weight gain of crossbred dairy cattle in grazing. R Bras Zootec.

[12] Backes AA, Paulino MF, Alves DD (2005). Body composition and energy and protein requirements of dairy crossbreeds and zebu bovines, castrated, in the growing and fattening phases. R Bras Zootec.

[13] Neves MLMW, Véras ASC, Souza EJO (2016). Energy and protein requirements of crossbred cattle in feedlot. Semin Cienc Agrar (Online).

[14] Prados LF, Valadares Filho SC, Detmann E (2015). Energy and protein requirements of ¾ Zebu × ¼ Holstein crossbreds fed different calcium and phosphorus levels in the diet. Arq Bras Med Vet Zootec.

[15] National Research Council (2000). Nutrient requirements of beef cattle.

[16] BRASIL, Ministry of Agriculture, Livestock and Food Supply (2000). Normative Instruction Nunber 3 of January 17th, 2000 [Internet]. Technical regulation on methods of desensitization for humanitarian slaughtering of animals.

[17] AOAC (1984). Official methods of analysis. Association of Official Analytical Chemists.

[18] Kobayashi K, Salam MU (2000). Comparing simulated and measured values using mean squared deviation and its components. Agron J.

[19] Tedeschi LO (2006). Assessment of the adequacy of mathematical models. Agric Syst.

[20] Backes AA, Paulino MF, Alves DD, Valadares Filho SC (2010). Relative size of the internal organs and gastrointestinal tract of dairy crossbreeds and zebu steers in fattening. Cienc Rural.

[21] Goulart RS, Alencar MM, Pott EB (2008). Body composition and protein and energy net requeriments of steers of four genetic groups finished in feedlot. R Bras Zootec.

[22] Prados LF (2012). Performance and nutritional requirements of cattle fed diets containing different levels of calcium and phosphorus [Dissertation].

[23] Silva GS, Véras ASC, Ferreira MA (2015). Performance and carcass yield of crossbred dairy steers fed diets with different levels of concentrate. Trop Anim Health Prod.

